# Hyperiid amphipods from the Gulf of Ulloa and offshore region, Baja California: The possible role of the gelatinous zooplankton as a transport vector into the coastal shelf waters

**DOI:** 10.1371/journal.pone.0233071

**Published:** 2020-11-05

**Authors:** Bertha E. Lavaniegos

**Affiliations:** Departamento de Oceanografía Biológica, Centro de Investigación Científica y Educación Superior de Ensenada, Baja California, México; Universidad de Antioquia, COLOMBIA

## Abstract

Hyperiid amphipod species from the Gulf of Ulloa, Baja California, and the adjacent region (from the shelf break to 200 km offshore) were analyzed to evaluate diversity and abundances. This productive area supports small-scale commercial fisheries, including sand bass (*Paralabrax nebulifer*), California spiny lobster (*Panulirus interruptus*), abalones, clams, and others. Strong coastal upwelling events were observed during summer seasons of the period 2002–2008 between Punta Eugenia and Punta Abreojos. The upwelling plumes at Punta Abreojos are transported southward in slope waters bordering the coastal shelf of the Gulf of Ulloa, contributing to the separation of coastal and oceanic regions, and explain differences in amphipod diversity and abundances between both regions. In the offshore region, the most abundant species were *Vibilia armata*, *Lestrigonus schizogeneios*, *Primno brevidens*, and *Eupronoe minuta*, similar to previous findings in northern regions of Baja California and southern California. However, abundances of these species were lower (10–30 individuals/1000 m^3^), only reaching 20–50% of abundance levels reported off northern Baja California. In the coastal shelf of the Gulf of Ulloa, amphipods were virtually absent during 2002, 2003 and 2006. However, during 2004 and 2005, abundances of *P*. *brevidens* increased (54 and 20 ind/1000 m^3^, respectively). Moreover, during the late summer of 2007, abundances of *L*. *schizogeneios*, *P*. *brevidens*, *Lycaea nasuta*, *Lycaea pulex*, and *Simorhynchotus antennarius* increased considerably (261, 39, 31, 68, 416 ind/1000 m^3^, respectively), indicating occasional utilization of the coastal shelf by pelagic amphipods. Changes in gelatinous populations (medusae, siphonophores, ctenophores, doliolids, and salps) paralleled changes in hyperiid populations, with highest abundances in 2005–2008 in the coastal shelf. Significant correlations of 17 amphipod species with gelatinous taxa, which are often used as host organisms by hyperiid amphipods, suggest that gelatinous presence enhanced hyperiid abundance and promoted the progression of hyperiid amphipods onto the coastal shelf during parts of the 2002–2008 period.

## Introduction

The zooplankton community has been intensively studied in the northern regions of the California Current System (CCS), but off Baja California it has received less attention, particularly in terms of taxa such as the hyperiid amphipods that comprise only a small proportion of the community and are therefore assumed to have minimal ecological importance. However, this perception may be incorrect for oceanic waters, where they are relatively abundant [1–4] and may represent attractive forage food for predators in the California Current such as small pelagic and myctophid fishes and seabirds [5–7]. Similar observations have been made in other eastern boundary upwelling ecosystems (EBUS), including the trophic roles of the amphipod *Themisto gaudichaudi* in the Benguela Current [8] and *Hyperia curticephala* in the Humboldt Current [9]. In the eastern tropical Pacific, some flying fishes and myctophids prey on hyperiids [10, 11]. Hyperiid amphipods have maximum abundances in high latitudes [12–15], while in tropical and subtropical regions they have low abundance but higher species diversity [16, 17]. However, more studies are required to quantify these crustaceans and their roles more precisely in trophic webs in tropical and subtropical seas.

Seasonal changes in hyperiid amphipod species assemblages have shown a strong coupling with upwelling dynamics off Oregon [18] and the southern sector of the CCS (southern California [19] and Baja California [1–2]). Lowest abundances occur in winter, followed by increases in spring-summer and maximum levels in autumn. However, hyperiids appear to avoid the zone of highest upwelling activity, as suggested by their relative scarcity in inshore waters compared to higher concentrations in the core and offshore part of the California Current [1, 18]. The oceanic abundances of hyperiids (<1 to 100 ind 1000 m-^3^ per species) are 50% higher than in the coastal shelf region [1]. The difference is striking because the main zooplankton species responsible for secondary production in Vizcaino Bay increase in neritic waters. The copepods *Calanus pacificus* and *Acartia tonsa* reach up to 138,000 and 60,000 ind 1000 m^-3^ respectively [20], and the euphausiid *Nyctiphanes simplex* reaches 35,000–87,000 ind 1000 m^-3^ [21, 22]. Mesoscale structures, particularly eddies, may contribute to concentrating the high phytoplankton productivity in the Gulf of Ulloa [23], where grazer organisms as copepods and euphausiids feed while amphipods are more carnivorous [24, 25]. The region also displays seasonal changes in circulation, with enhanced nearshore poleward flow from July to October [26]. This pattern is also observed in other EBUS [27] and is responsible for transporting oceanic fauna onto the shelf areas. As a consequence of such dynamic circulation, productivity is high, similar to other EBUS [28, 29], making the region a hotspot for whales [30], sea turtles [31, 32], tuna [33], and swordfish [34], which currently supports a successful artisanal coastal fishery out of Punta Abreojos [35, 36]. Understanding the zooplankton community dynamics in this area can help improve the management of these small-scale fisheries.

In order to expand our understanding of zooplankton community and mechanisms influencing their variability in upwelling ecosystems of subtropical latitudes comparable to the Gulf of Ulloa and offshore region, the present research describes interannual variability in summer species composition of hyperiid amphipods during 2002–2008. The study specifically explores (1) the distribution and abundance of hyperiid amphipods, by assessing interannual variability in species assemblages; and (2) investigating the correlation between hyperiids and gelatinous zooplankton. This study presents evidence of intermittent occupation by hyperiids of the coastal shelf in the Gulf of Ulloa, Baja California, and possible mechanisms underlying this behavior.

## Materials and methods

### Study area

**Fig 1:**
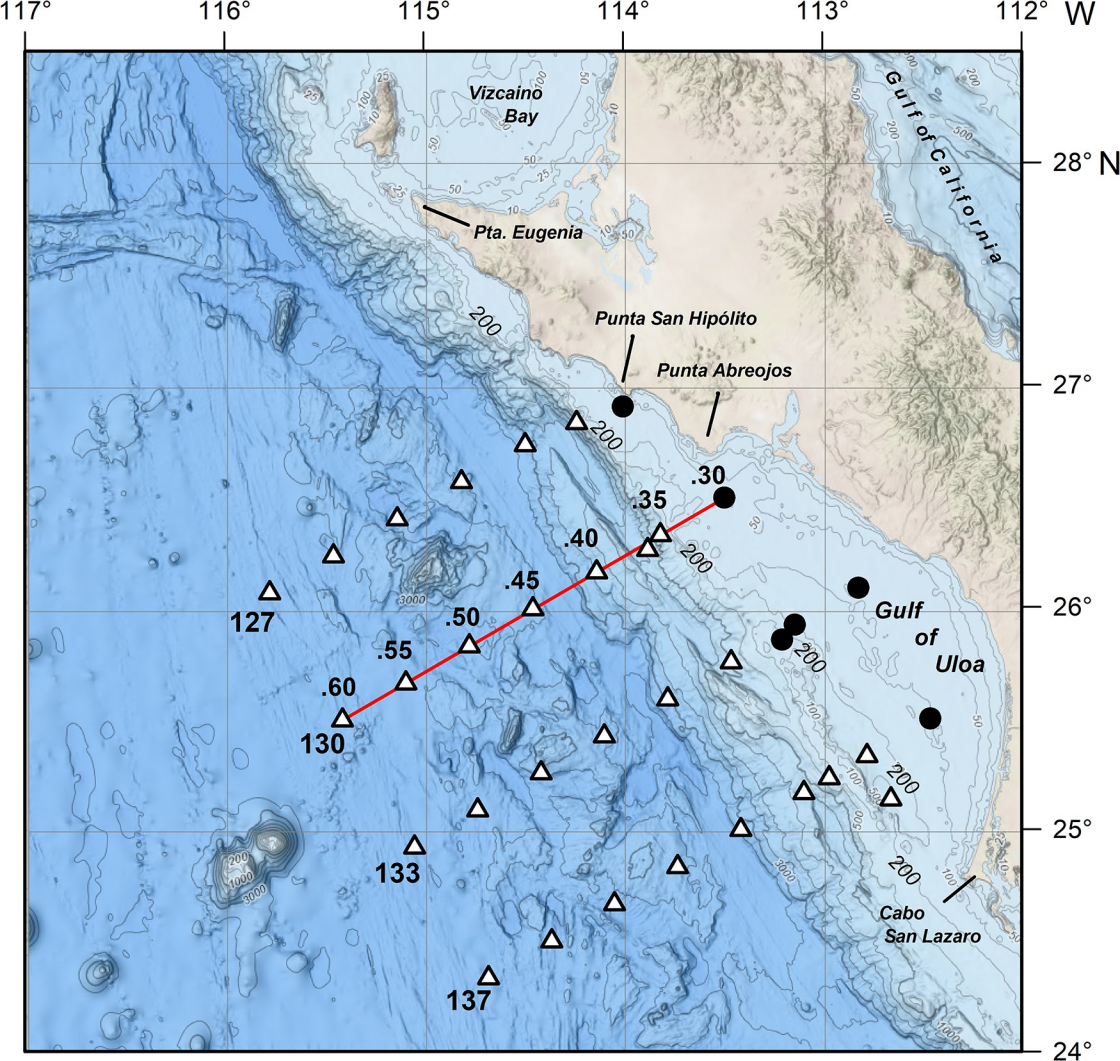
Study area. Gulf of Ulloa and offshore region showing the sampling grid (symbols) and bathymetry (m, shaded blues). Stations sampled varied by cruise (see S1 Table). The 200 m isobath separates coastal from oceanic stations (round and triangle symbols, respectively). Red solid line indicates the transect used in vertical profiles. Numbers are transect-lines and stations in decimals are indicated for Line 130. Topographic chart from GEBCO (https://www.gebco.net/data_and_products/gridded_bathymetry_data/gebco_2019/gebco_2019_info.html).

The study area is located off southwest Baja California between Punta Eugenia and Cabo San Lazaro and is characterized by a relatively wide coastal shelf (Fig 1). The coastline is oriented NW-SE, favorable for upwelling [37], which is further reinforced by the narrow step-shelf between Punta Eugenia and Punta Abreojos. Farther south, the coastal shelf broadens to form the embayment named the Gulf of Ulloa (GU). Upwelling events induced by trade winds (Easterlies) are particularly frequent during spring and summer [38]. The lowest sea surface temperature (SST) occurs in February–April (18°C) while the maximum is recorded in late summer (25°C), associated with a seasonally enhanced poleward current [26]. Poleward flow produces a decrease in chlorophyll during summer months, contrasting with the high productivity in spring (250–750 mg C m^-2^ d^-2^), when equatorward flow and strong upwelling dominate [23].

### Sampling

The sampling stations were arranged in four transect-lines perpendicular to the coast (Fig 1) from seven cruises performed every summer from 2002–2008 by the IMECOCAL program (Spanish acronym of: *Mexican Investigations of the California Current*). At each station, hydrographic data were recorded with a CTD (Seabird Electronics Inc., 9/11). Zooplankton sampling was carried out by oblique tows of a bongo net (71 cm-diameter, 505 μm mesh width) with a digital mechanical flowmeter. In the epipelagic oceanic region, tows were conducted from 0–210 m, while at stations located in the coastal shelf the tow depth was from the surface to 10–15 m above to the bottom. The samples were preserved with 4% formalin.

Only the nighttime samples were selected to perform taxonomic analysis of hyperiid species in the offshore region to reduce variability due to vertical migration, as has been reported for some hyperiid species [see 39, 40]. However, for the coastal shelf (bottom depth < 200 m) daytime samples were also included due to the low number of stations. The cruise dates and number of samples analyzed are shown in Table 1 (details of sampling stations in S1 Table).

**Table 1:** IMECOCAL cruises with dates and number of zooplankton samples used in taxonomic identification. Cruise dates correspond to the area considered in the present study (see Fig 1).

Cruise	Date	Number of samples		
		Offshore region	Gulf of Ulloa	
		N	D	N
**0207**	26 Jul–1 Aug 2002	10	2	3
**0307**	23–27 Jul 2003	8	3	1
**0407**	23–29 Jul 2004	8	3	3
**0507**	29 Jul–4 Aug 2005	10	4	2
**0607**	19–24 Jul 2006	8	1	4
**0709**	6–13 Sep 2007	12	4	2
**0807**	14–21 Jul 2008	14	3	3

The sampling hour is indicated as daytime (D) and nighttime (N).

### Taxonomic analysis

Hyperiid species were identified from the complete sample, based on taxonomic keys [16, 41]. Additional counting of potential gelatinous hosts of amphipods [14, 42, 43] were made using a fraction of the sample (1/8 or 1/16). The gelatinous organisms were identified only to major groups (medusae, siphonophores, ctenophores, doliolids, salps) via imaging [44, 45].

In the present study, hyperiid species occurring in >55% of samples are considered dominant, those in 31–55% are common, those in 10–30% are sparse, and those in <10% are rare, following definitions from [46]. The geometric mean (GM) is used instead of numerical mean because it is less affected by particularly small or large values and data distribution skew.

### Statistical analysis

To provide a context of seasonal changes in amphipod abundance and distribution, data from all seasons in 2005 were included to produce a full annual cycle. For interannual analyses (summer hyperiids during 2002–2008), zooplankton stations were separated into onshore or offshore regions, as demarcated by the 200 m isobath (Fig 1). For each region, tnterannual comparisons of the most abundant species were done with the Kruskal-Wallis test. To determine the specific years with differences, *a posteriori* comparison with the Tukey test was made. Interannual comparisons were also made for gelatinous zooplankton groups.

Multivariate cluster analysis was used to address community structure across years. Summer 2007 was excluded in this analysis due to sampling bias (September cruise, compared to Jul-Aug cruises in other years). To explore amphipod community grouping by stations, hierarchical cluster analysis was performed with STATISTICA 7.1. Euclidian distances were calculated using transformed data (log10 [x-+1]) to weight the contribution of abundant and rare species. The data matrix consisted of 70 species × 83 samples, after exclusion of 19 rare species (occurring in only one sample) and 7 samples from coastal stations without amphipods. Clusters were defined with the Ward´s linkage hierarchical method [47, 48]. Further, analysis of similarities (ANOSIM) was done to test the hypothesis of differences between clusters based on a resemblance matrix of Bray-Curtis index with the software PRIMER-7. The resemblance matrix was also used to estimate percentages of similarity (SIMPER) to determine the contribution to similarity of each species in the clusters [49].

The potentially symbiotic relationship between hyperiid species and gelatinous zooplankton groups was analyzed using Spearman correlation analysis of all summer samples (N = 101).

### Physical variables and climate index

Summer profiles of temperature and salinity were made for the transect-line 130 in the upper 200 m. Distribution of temperature at 10 m depth was used in contour maps to relate to amphipod assemblages. The climatic context was provided by the Oceanic El Niño Index (ONI) from the region 3.4 (5°N-5°S, 120°-170°W), available at (https://origin.cpc.ncep.noaa.gov/products/analysis_monitoring/ensostuff/ONI_v5.php), and temperature anomalies in the study area during 2002–2008. The anomalies were estimated by removing climatic means from a prior period (1951–1966) using data collected by the California Cooperative Oceanic Fisheries Investigations (www.calcofi.org).

## Results

### Physical environment

**Fig 2:**
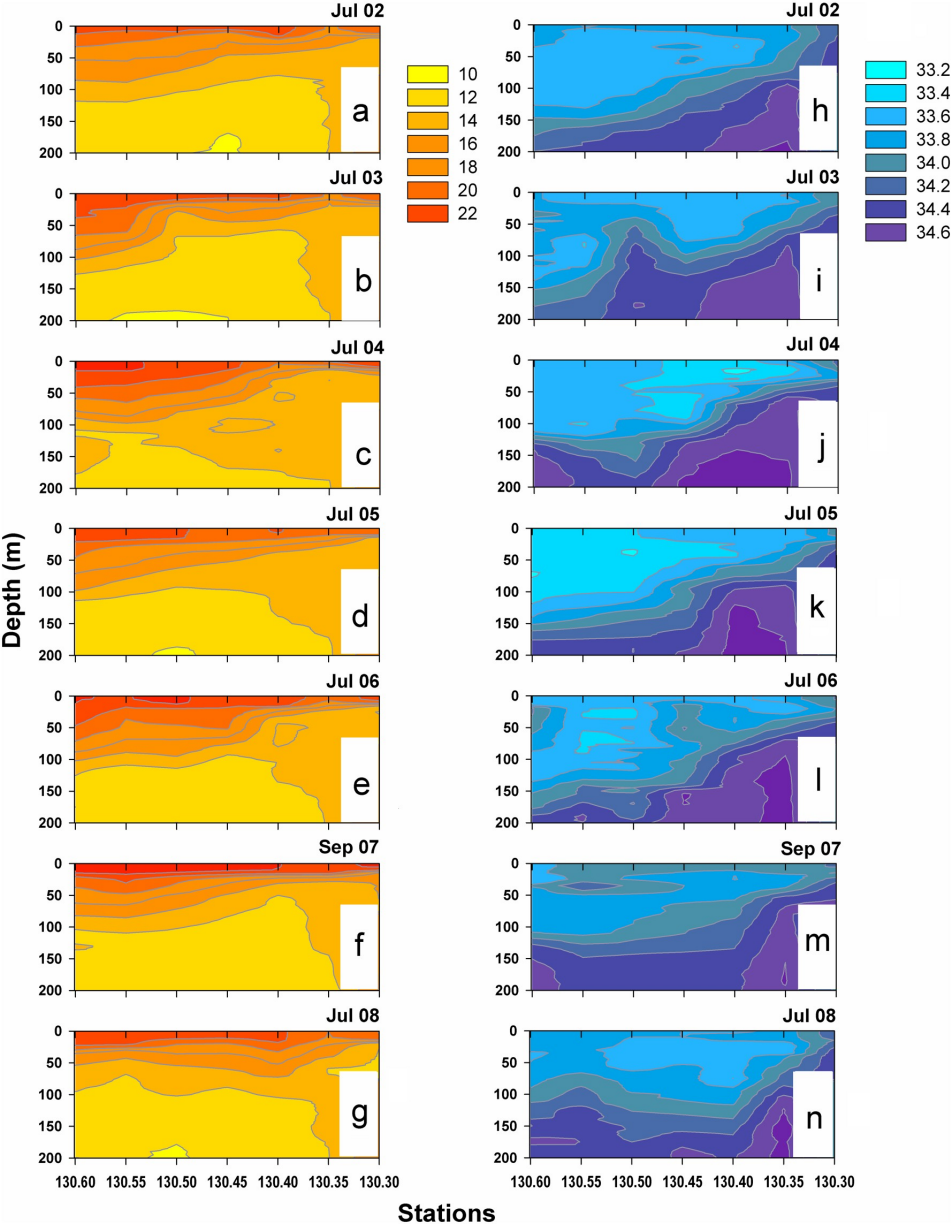
Vertical profiles. Temperature (a–g) and salinity (h–n) in the upper 200 m along the line 130 during the summers of 2002–2008.

Vertical sections of temperature along Line 130, extending from Punta Abreojos to 250 km offshore (Fig 2a–2g), showed strong stratification during summer. Sea surface temperatures were slightly lower in 2002, 2005, and 2008, ranging from 19–22°C offshore and 18–20°C inshore. Maximum SST values were recorded in 2007 (22–23°C) because sampling was conducted in September (Fig 2f), when SST increases compared to mid-summer, the period when all other years were sampled. The upwelling footprint throughout the study period was more evident in station 130.35, slightly farther offshore, than in the most coastal station 130.30. Despite low SST at Station 130.35, temperature at 100–200 m depth was consistently higher than the rest of stations in Line 130 due to the influence of the California Undercurrent, transporting warm Pacific Equatorial Water poleward.

A high salinity core in stations 130.35 and 130.40 also indicates the influence of the California Undercurrent (Fig 2h–2n). The tilting of the isohalines is pronounced closest to the coast, indicating intense upwelling activity. In July 2002 and again in summers 2007 and 2008, high-salinity upwelled water (>34 psu) reached the coastal shelf, but from 2003–2006 low–salinity water in the upper layer masked upwelling.

In summary, thermohaline conditions off southern Baja California showed marked onshore-offshore differences during every summer season, and evidence for intense upwelling activity in the slope region (Stn. 130.35). The main interannual differences were slightly lower SST in 2002, 2005, and 2008 compared to other summers, and low salinity during 2002–2006.

**Fig 3:**
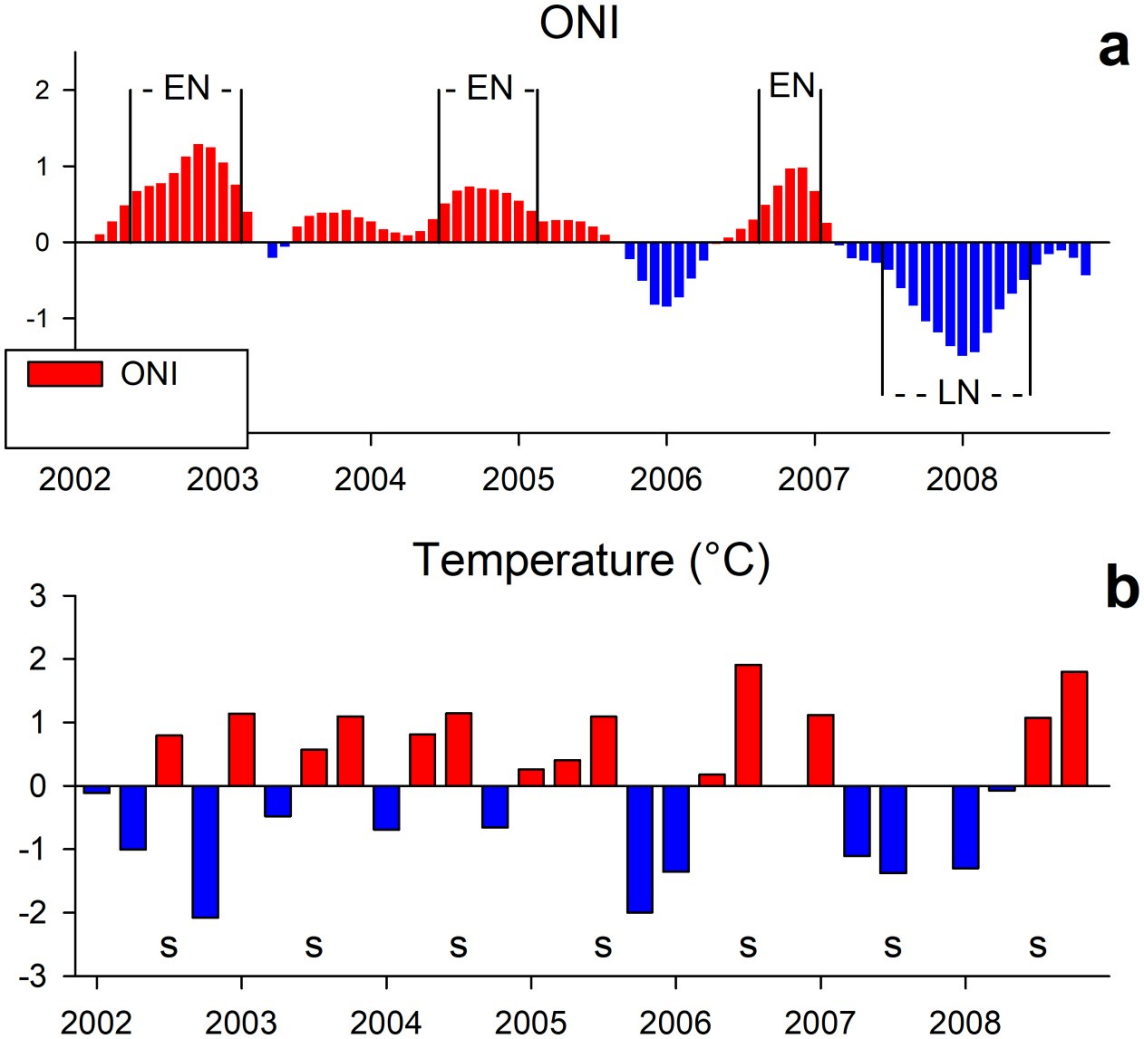
Climatic context during 2002–2008. Pacific basin conditions as indicated by the Oceanic El Niño Index (ONI) from region 3.4 (a), and surface temperature anomalies in the study region (b). Letter S indicate the summer.

Interannual climatic variability in the Pacific Ocean during 2002–2008 showed the occurrence of three weak El Niño events, in 2002–2003, 2004–2005, and 2006–2007 (Fig 3a). Those events had short duration, but two of the summer surveys were performed within some of these events: July 2002 (0207) during El Niño 2002–2003, and July 2004 (0407) during El Niño 2004‒2005. The survey of September 2007 (0709) occurred during La Niña 2007–2008. Sea surface temperature anomalies in the study region (Fig 3b) aligned with the ENSO cycle, negative during the ENSO cool phase and positive during the warm phase. The exception to this pattern was the negative anomaly at the end of 2002, related to an intrusion of Subarctic Water described elsewhere [50, 51].

### Distribution and abundance of hyperiid amphipods

**Fig 4:**
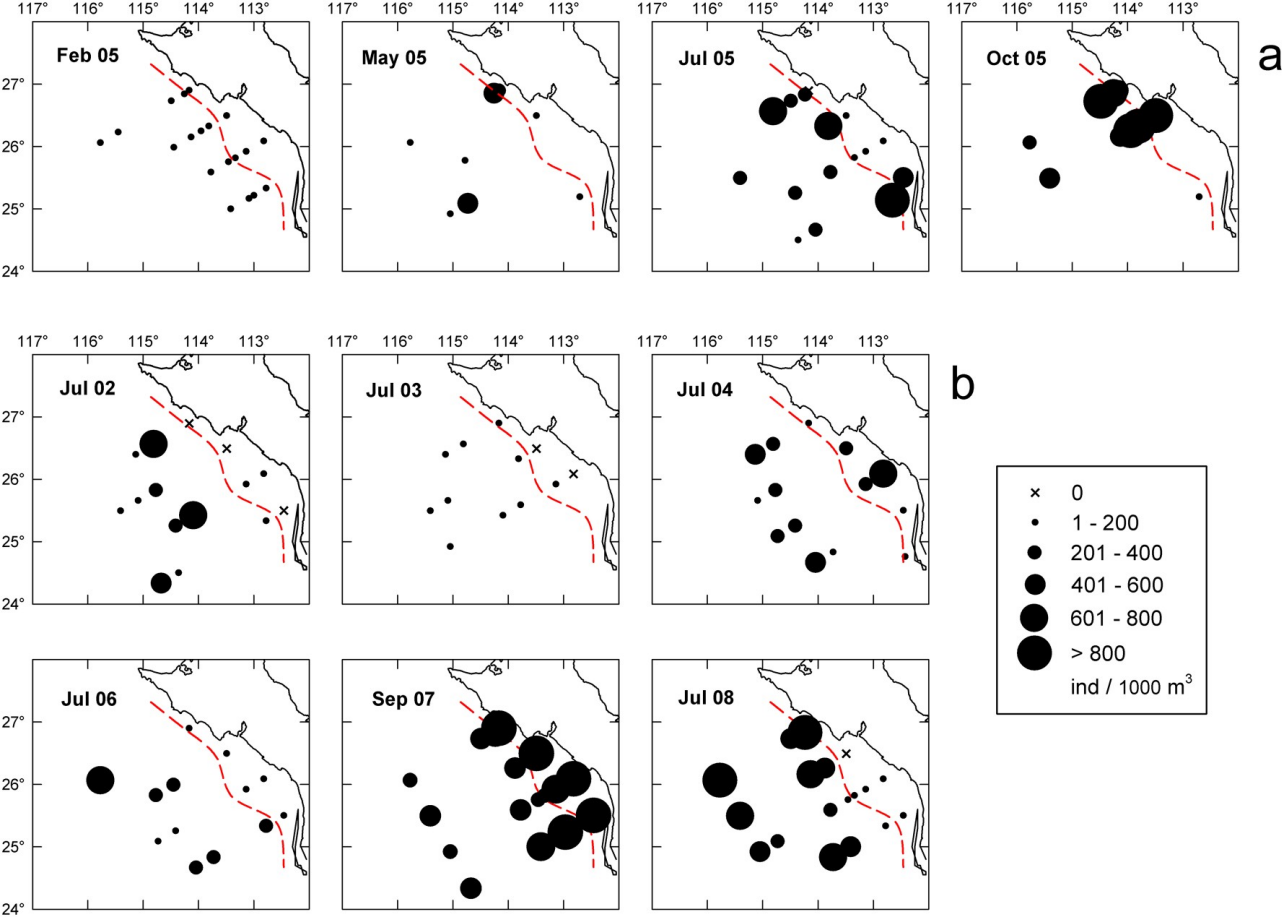
Distribution of hyperiid amphipods. Total amphipods distribution in the Gulf of Ulloa and offshore region during the annual cycle 2005 (a) and the summers of 2002–2008 (b). Red dashed line indicates the onshore/offshore station divide.

**Fig 5:**
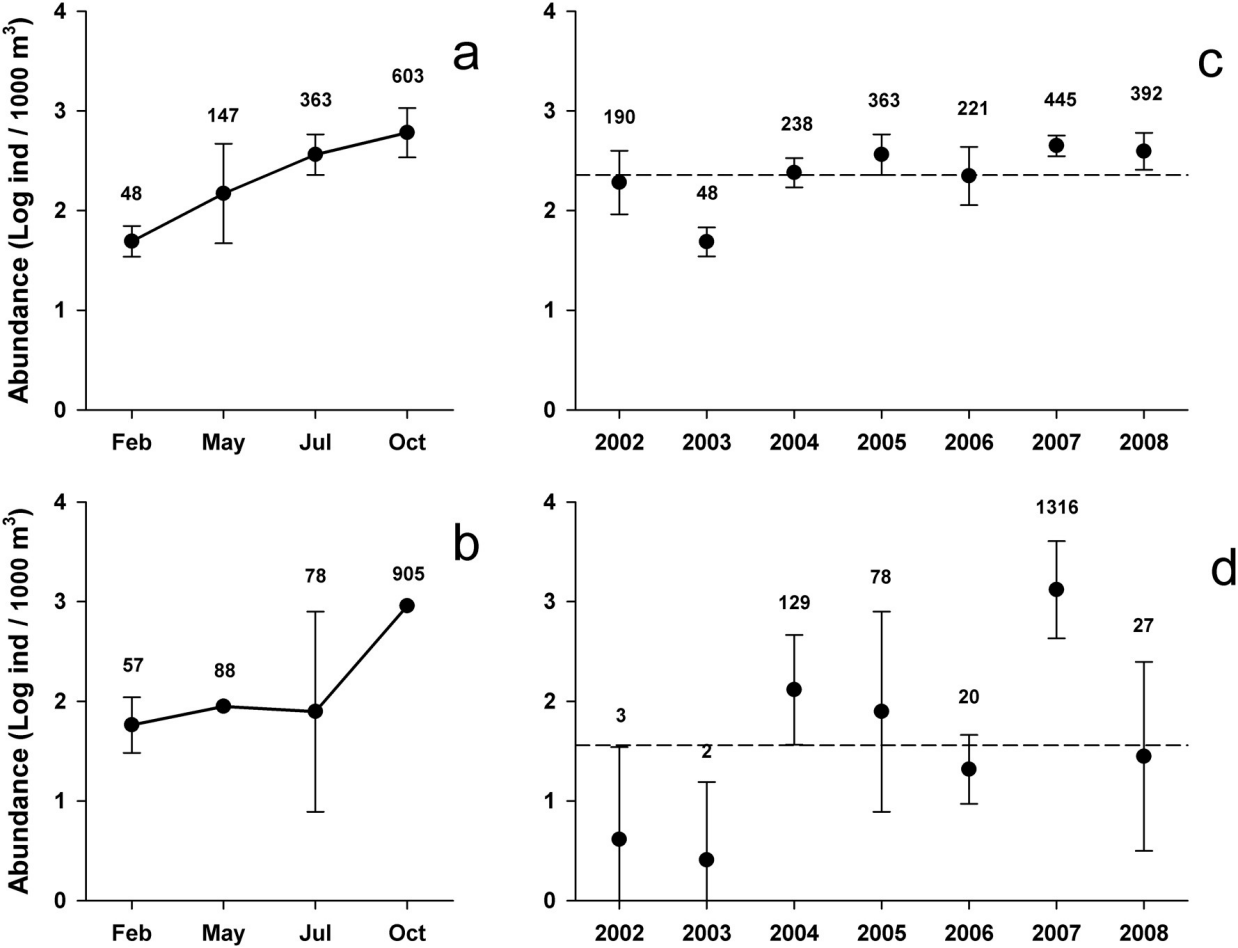
Abundance of hyperiid amphipods. Mean (± 95% confidence interval) of total hyperiids in the oceanic region off southern Baja California (a, c) and the Gulf of Ulloa (b, d), during the annual cycle 2005 (a–b) and the summers of 2002–2008 (c–d). Data transformed with log_10_[x+1]. The summer long-term mean is indicated by the dashed line; numeric values express geometric means.

First examining seasonal changes in hyperiid amphipods in 2005, abundances were low in February (< 200 ind/1000 m^3^) but homogeneously distributed across the GU and offshore region (Fig 4a). Low abundances persisted in spring, apart from three stations with 326–541 ind/1000 m^3^, located in the coastal shelf, the shelf break, and oceanic region (Stns. 127.35, 127.36, 133.55, respectively). A regionwide increase in abundance occurred in oceanic waters in July, while low abundances persisted in the neritic region. By October 2005, high abundance (> 500 ind/1000 m^3^) was observed near the coast and slope waters (Fig 4a). Therefore, hyperiid amphipods showed a gradual increase from winter to fall in the offshore region (Fig 5a) while the increase in the GU was limited to autumn (Fig 5b). However, the number of samples from the coastal shelf during May and October 2005 was too low to establish a more robust conclusion.

Turning to interannual comparisons of summer abundances, total hyperiid amphipods varied widely in samples, from 0 to 3,732 ind/1000 m^3^. Except for 2007, hyperiids collected at GU had low abundance (< 150 ind/1000 m^3^) and were absent in seven samples (3 in 2002, 2 in 2003, 1 each in 2005 and 2008; Fig 4b). Approximately 26% of all the GU samples had abundances higher than 200 ind/1000 m^3^ in 2004, 2005, and 2007, and four stations in 2007 surpassed 1000 ind/1000 m^3^. Thus, except for late summer 2007, amphipods showed a clearly oceanic tendency, indicated by higher presence and abundance in offshore stations with bottom depth >200 m. In this offshore region, the highest abundances were observed in 2007 and 2008, and the lowest in July 2003 (Fig 4b).

The Kruskal-Wallis test comparing summer abundances across years in the offshore region was significant, and summer 2003 was notably lower than all other years (H = 27.6, p < 0.001). The GM in 2003 was 48 ind/1000 m^3^; it fluctuated between 190 and 445 ind/1000 m^3^ in the other years (Fig 5c). In the GU, hyperiid abundance also presented significant interannual differences (H = 24.6, p < 0.001). The highest abundance recorded in 2007 in the GU (GM = 1,316 ind/1000 m^3^) was significantly higher than for 2002, 2003, 2006, and 2008, when the GM ranged from 2 to 27 ind/1000 m^3^ (Fig 5d). The abundance in 2004 (GM = 129 ind/1000 m^3^) was also significantly higher in relation to 2002 and 2003 (p = 0.020 and 0.010, respectively); 2005 (GM = 78 ind/1000 m^3^) had significantly higher abundance than only 2003 (p = 0.036).

#### Interannual variability of hyperiid species

Hyperiid amphipod diversity was high in the study region: 91 species were recorded in the 2002–2008 period (S2 Table). Eighty-eight of these species occurred in the oceanic region, while 56 were in neritic waters. There were five dominant species in the whole region (frequency 58–80%; abundance GM 4–15 ind/1000 m^3^). All dominant species pertained to the infraorder Physocephalata, with one in the superfamily Vibiloidea (*Vibilia armata*), two in the superfamily Phronimoidea (*Lestrigonus schizogeneios* and *Primno brevidens*), and two in the superfamily Platysceloidea (*Eupronoe minuta* and *Simorhynchotus antennarius*).

There were 12 common species (frequency 31–55%), with one from Physosomata (*Scina tullbergi*) and the rest from Physocephalata (*Paraphronima gracilis* of the superfamily Vibiloidea, 6 species of Phronimoidea, and 3 in Platysceloidea. The GMs of these species ranged from 0.6–2.6 ind/1000 m^3^ (S2 Table).

Sparse species (frequency 10–30%) occurred with GMs of 0.1–0.9 ind/1000 m^3^. They included one species from Physosomata (*Scina borealis*) and 32 from Physocephalata, spread over the superfamilies Vibiloidea (12%), Phronimoidea (28%), and Platysceloidea (59%). The complete list of species is shown in S2 Table. Almost all sparse species were missing in one or more years, but a few occurred across the period 2002–2008 (*S*. *borealis*, *Lycaeopsis themistoides*, *Pronoe capito*, and *Rhabdosoma whitei*).

A high number of species were categorized as rare (<10% in samples): 7 from Physosomata and 36 from Physocephalata. Their global GM was below 0.1 ind/1000 m^3^ (S2 Table). The low abundance of some rare species is probably due to their meso- and bathypelagic distributions, meaning they are rarely captured in the upper layer. Such is the case for *Lanceola clausi*, *Scypholanceola aestiva*, and *Scina curvidactyla* [16]. Most of the rare species only occurred in a single year (51%) and some occurred in only a single sample (42%). The year with the lowest number of rare species was 2003, and the highest was 2007, at 5 and 51% respectively. The remaining years showed values between 23–35%. Most of the rare species were found in the offshore region (72%); only 7% occurred exclusively inside the GU, and the remaining 21% occurred in both regions.

**Fig 6:**
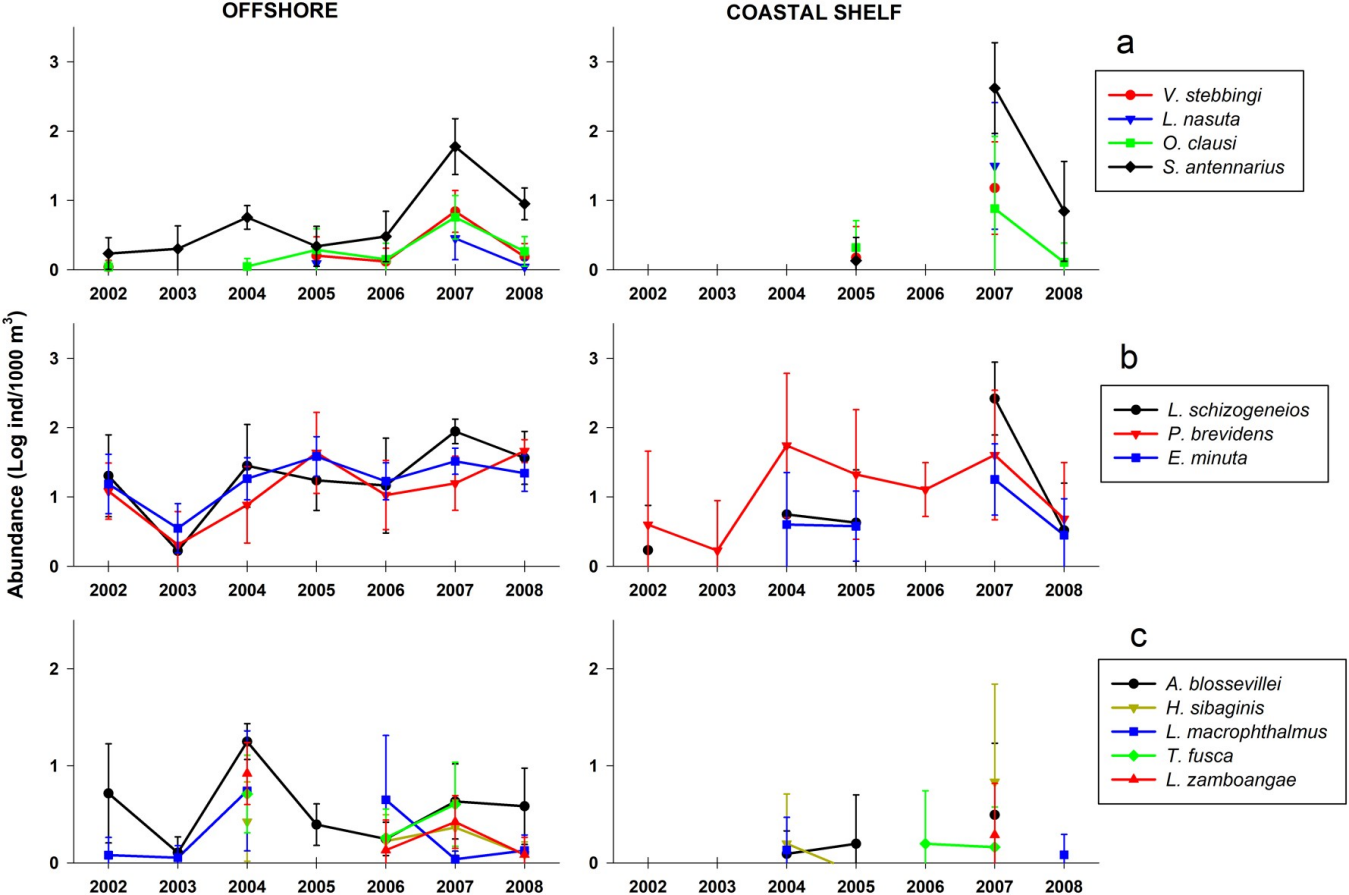
Patterns of interannual variability. Mean (± 95% confidence interval) in species groups showing an increase in 2007 (a), decreasing in 2003 (b), or increasing in 2004 (c).

Interannual abundance comparisons within non-rare species were significant in 26 cases in the offshore region, representing 80% of dominant species, 67% of common species, and 45% of sparse species (p<0.01, Table 2). A recurrent pattern was the high abundance during 2007, significantly higher than all other years, depicted for *S*. *antennarius*, *Vibilia stebbingi*, *Lycaea nasuta*, and *Oxycephalus clausi* (Fig 6a). In other species as *S*. *tullbergi* and *Hyperoche medusarum*, the abundance was similarly high in 2007 and 2008 compared to 2006. In *Laxohyperia vespuliformes* the abundance was high in 2007 and 2005 compared to 2002–2004 (see S1–S3 Figs).

**Table 2:** Interannual comparisons of hyperiid species. Abundances of dominant, common, and sparse species compared across years using the Kruskal-Wallis test.

Species	H	p	Multiple comparisons
**Offshore region** (N = 70)			
*Amphithyrus sculpturatus*	33.4	<0.001	(2007,2008) < (2002,2004,2005) 2003 < 2002
*Anchylomera blossevillei*	21.9	0.001	(2003,2005,2006) < 2004
*Eupronoe armata*	21.5	0.002	(2002,2003,2005,2008) < 2007
*Eupronoe maculata*	21.3	0.002	(2003,2004,2005) < 2002
*Eupronoe minuta*	20.2	0.003	2003 < other years
*Hyperioides longipes*	30.3	<0.001	other years < 2005
*Hyperioides sibaginis*	19.1	0.004	(2002,2005) < 2004
*Hyperoche medusarum*	17.4	0.008	2006 < (2007,2008)
*Laxohyperia vespuliformis*	28.5	<0.001	(2002,2003,2004) < (2005,2007) 2006 < 2007
*Lestrigonus bengalensis*	19.4	0.004	2002 < (2004,2005,2008)
*Lestrigonus macrophthalmus*	19.4	0.004	(2002,2003.2005,2007,2008) < 2004 (2005,2007) < 2006
*Lestrigonus schizogeneios*	23.3	<0.001	2003 **<** (2002,2004,2005,2007,2008)
*Lycaea nasuta*	27.1	<0.001	other years < 2007
*Lycaea serrata*	17.7	0.007	NS
*Lycaeopsis zamboangae*	38.3	<0.001	other years < 2004 (2002,2005) <2007
*Oxycephalus clausi*	26.4	<0.001	other years < 2007
*Phronima curvipes*	20.2	0.003	(2003,2004,2007) < 2008
*Phrosina semilunata*	24.6	<0.001	2007 < (2005,2006,2008) 2003 < 2005
*Platyscelus ovoides*	25.7	<0.001	(2003,2004) < 2002
*Primno brevidens*	24.0	<0.001	2003 **<** (2005,2006,2008)
*Scina tullbergi*	39.5	<0.001	(2002,2003,2004,2006) **<** (2007,2008)
*Simorhynchotus antennarius*	37.7	<0.001	other years **<** 2007 (2002,2003,2005) < 2008
*Themistella fusca*	31.6	<0.001	(2002,2003,2005,2008) < (2004,2007)
*Tryphana malmi*	18.4	0.005	other years < 2008
*Vibilia stebbingi*	33.7	<0.001	other years < 2007
*Vibilia viatrix*	25.6	<0.001	(2003,2005,2006,2007,2008) < 2002
**Coastal shelf** (N = 38)			
*Eupronoe minuta*	19.9	0.003	(2002,2003,2006) < 2007
*Laxohyperia vespuliformis*	16.9	0.010	(2002,2004,2006) < 2007 2004 < 2005
*Lestrigonus bengalensis*	23.4	<0.001	other years < 2007
*Lestrigonus schizogeneios*	21.6	0.002	other years < 2007
*Lycaea nasuta*	29.7	<0.001	other years < 2007
*Lycaea pulex*	20.6	0.002	other years < 2007
*Phronima atlantica*	24.8	<0.001	other years < 2007
*Platyscelus ovoides*	18.4	0.005	(2002,2003,2004,2006,2008) < 2007
*Rhabdosoma whitei*	12.0	0.010	other years < 2007
*Simorhynchotus antennarius*	30.1	<0.001	other years < 2007 (2002,2003,2004,2006) < 2008
*Tryphana malmi*	23.2	<0.001	(2002,2003,2004,2005,2006) < 2007
*Vibilia stebbingi*	25.9	<0.001	other years < 2007

Only species with significant results are shown (α < 0.01). Specific years with significant differences are indicated, which resulted from Tukey multiple comparisons.

In general, 2007 and 2008, and to a lesser extent 2004, appeared to be years of high abundance for many species, while 2002, 2003, and 2006 were years of low abundance. Some species exhibited a uniform pattern throughout the entire study period except for a strong decrease in 2003, particularly remarkable in the dominant species *L*. *schizogeneios*, *P*. *brevidens*, and *E*. *minuta* (Fig 6b). Summer 2004 was notable for high abundances *of Anchylomera blossevillei*, *Hyperioides sibaginis*, *Lestrigonus macrophthalmus*, *Themistella fusca*, and *Lycaeopsis zamboangae* (Fig 6c).

In the coastal shelf region, 12 species showed significant interannual differences (p<0.01, Table 2). In all cases, abundances were highest in 2007 and generally lowest in 2002, 2003, and 2006. During 2007, *Lestrigonus bengalensis*, *L*. *schizogeneios*, and *S*. *antennarius* reached higher GM within the coastal shelf (28, 261, and 416 ind/1000 m^3^, respectively) compared to the offshore region (3, 87, and 59 ind/1000 m^3^; Fig 6 and S2 and S3 Figs).

#### Assemblages of hyperiid species

**Fig 7:**
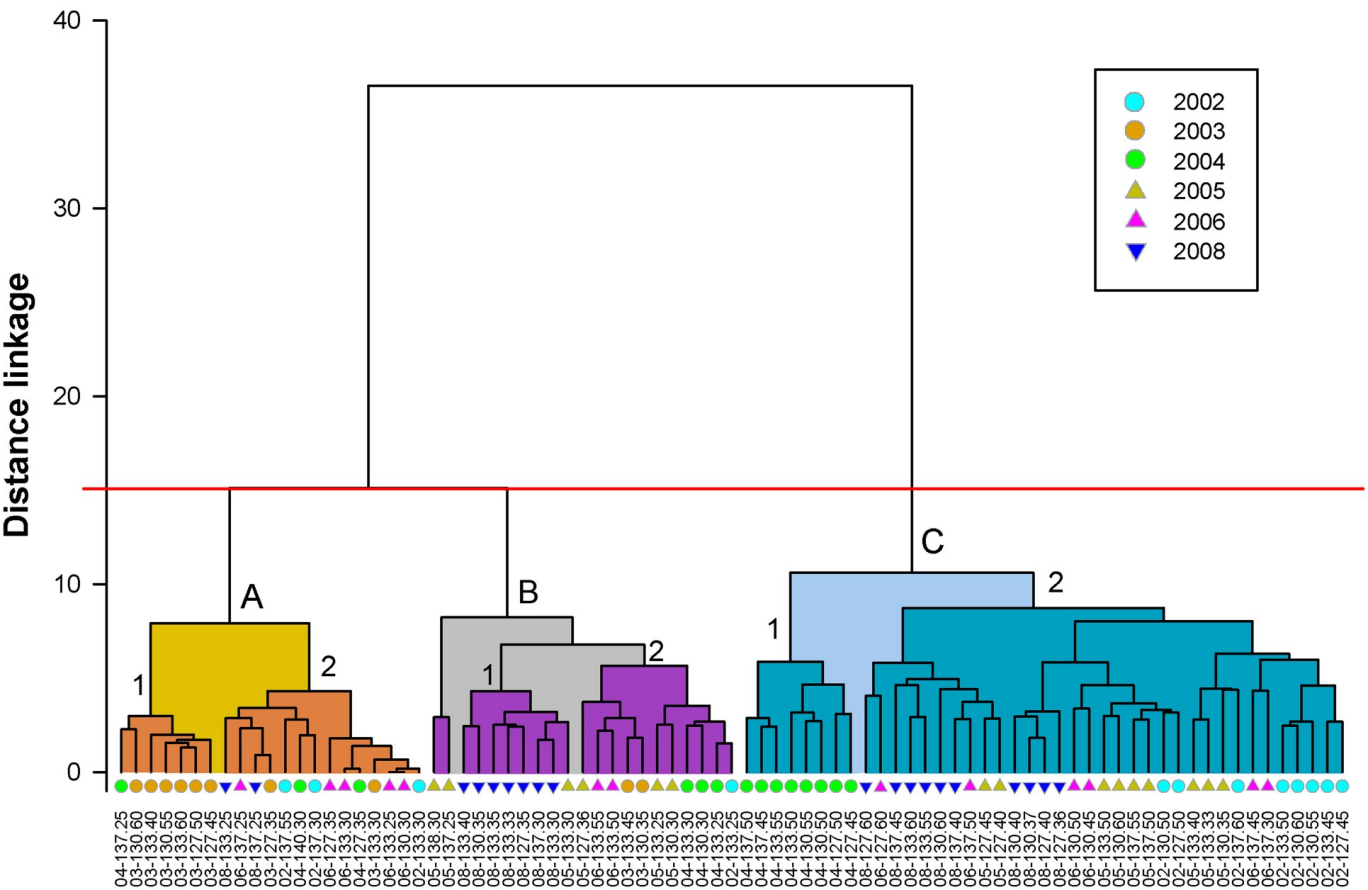
Hyperiid assemblages. Clustering of sampling stations based on abundances of 75 species. Clusters formed at a distance linkage of 15 (cutoff line in red) designed by letters (A, B, C), and subgroups by numbers. In the x-axis the sampling stations are shown with symbols in color indicating the year.

**Fig 8:**
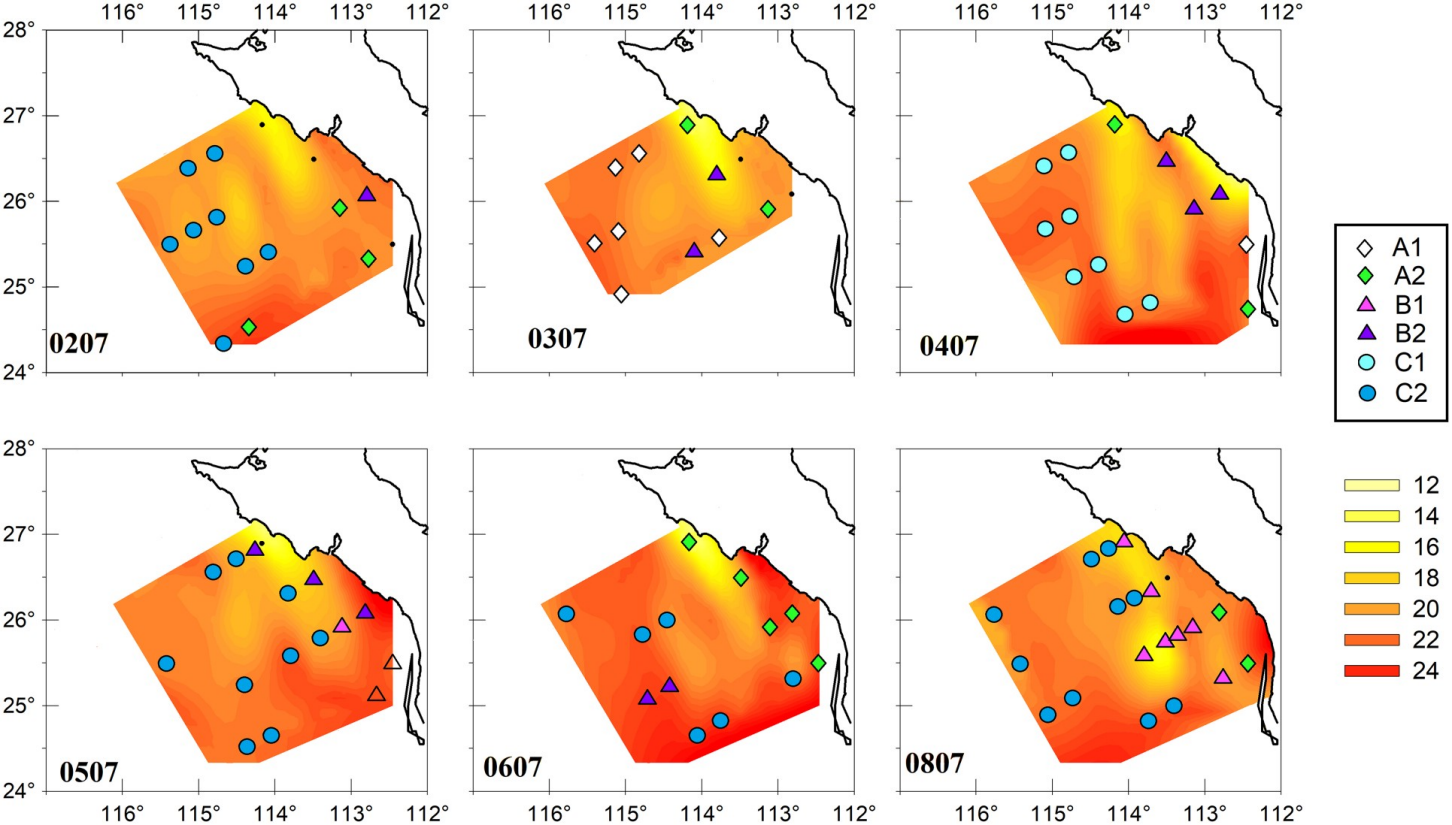
Geographic distribution of clusters. Clusters defined in Fig 7 are shown and sea surface contours (°C) were added as a reference to climatic conditions. Uncolored triangles pertain to cluster B (no subgroup), and black points indicate stations without amphipods.

Multivariate analysis excluding 2007 resulted in three main clusters with variable number of stations (indicated with letters A–C in Fig 7). Cluster A may be split into two subgroups, one mainly oceanic (A1) and other neritic (A2). The oceanic stations in the A1 assemblage were from 2003, along with one coastal station from 2004 (137.25). Assemblage A2 contained two oceanic stations from 2002 (137.30, 137.55) and coastal stations from all years except 2005 (Fig 8). Cluster B was also dominated by coastal stations and is divided into two subgroups of different years: B1, which consists of 8 stations (seven from 2008, and one from 2005), and B2, which has a mixture of eleven stations from 2002–2006. One pair of stations from 2005 (137.25, 138.30) were not in subgroup B1 or B2 but formed a part of Cluster B (Fig 7). The largest oceanic cluster, C, consisted of 41 stations, split in two subgroups: C1, with samples only from 2004; and C2, including 33 samples from 2002, 2005, 2006, and 2008 (Figs 7 and 8).

The differences among the six clusters, including groups and subgroups, were confirmed with ANOSIM, which had a global R = 0.654 (p = 0.001). The pairwise tests between clusters also showed significant differences in all cases (p < 0.007).

**Fig 9:**
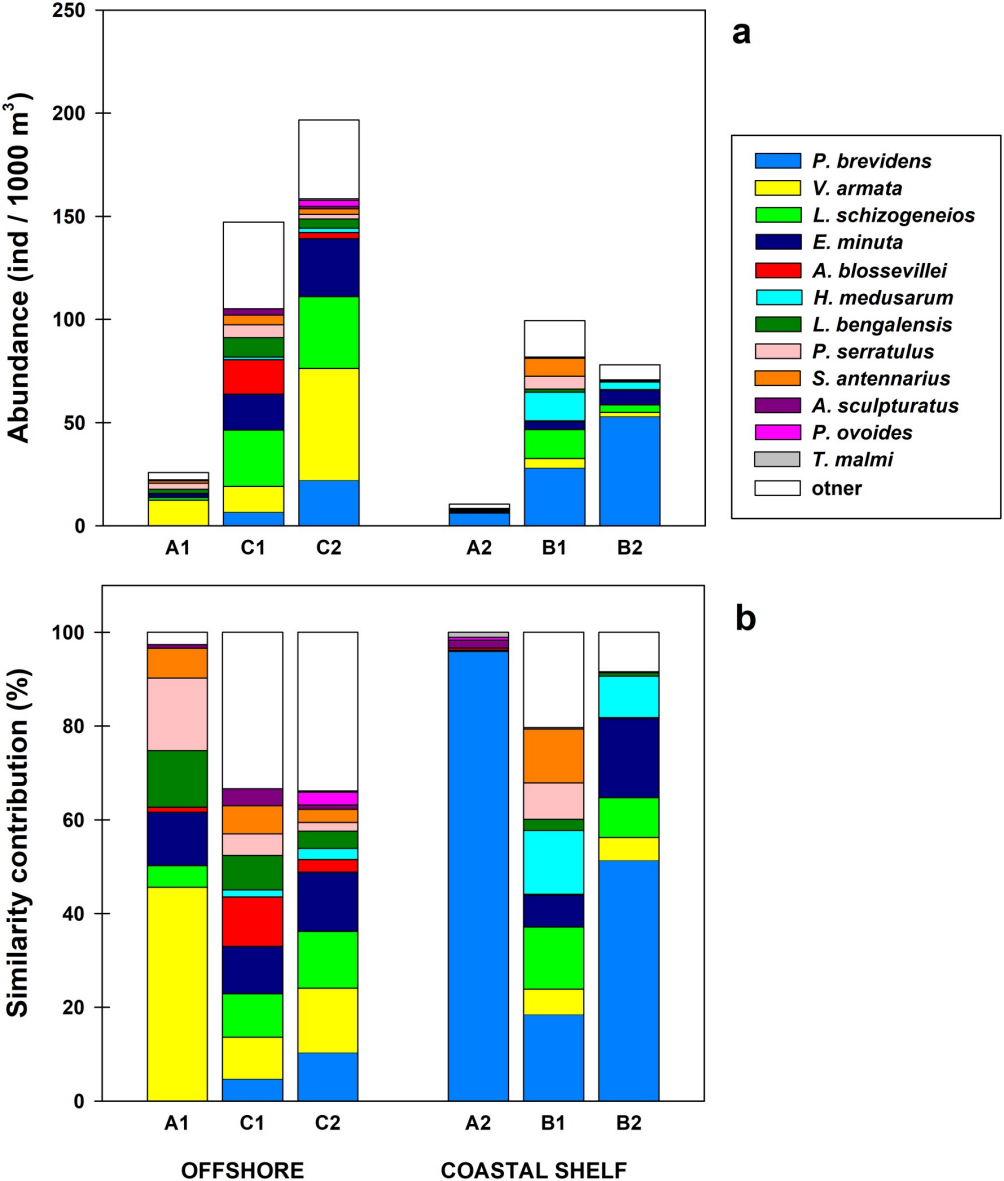
Species contribution in clusters. Stacked geometric means (a) and contribution to similarity (b) of main species in clusters defined in Fig 7. The selected species are a combination of the four with highest similarity in each cluster.

The clusters considered coastal showed a strong contrast in species composition between Cluster A2 and the other two clusters (B1 and B2, Fig 9). Besides different species composition, Cluster A2 had low amphipod abundance. This cluster was dominated by samples from 2006 (Fig 8). Clusters A2 and B2 showed the highest contribution to similarity by *P*. *brevidens*, with 95.9 and 51.3% respectively (Fig 9b). However, the high percentage of this species in cluster A2 was due to a low diversity (22 species with abundances <0.6 ind/1000 m^3^); *P*. *brevidens* also had low abundance (GM = 6 ind/1000 m^3^) compared to the other coastal clusters. In contrast, the assemblage B2 had abundant *P*. *brevidens* (GM = 53 ind/1000 m^3^) and included33 other species, though only six (*E*. *minuta*, *H*. *medusarum*, *L*. *schizogeneios*, *V*. *armata*, *Phronima sedentaria*, and *S*. *borealis*) were moderately abundant (1–7 ind/1000 m^3^). The assemblages of hyperiid species in clusters A2 and B2 represent typical neritic communities since they consist of data from various years, even though B2 was more characteristic of 2004 and 2005 (Fig 8). The cluster B1 included coastal and shelf break stations from 2008, with most abundant species *P*. *brevidens*, *L*. *schizogeneios*, and *H*. *medusarum* (GMs >10 ind/1000 m^3^; Figs 8 and 9a). These three species combined contributed 45.4% to similarity in cluster B1 (Fig 9b).

In the offshore region, the interannual separation within different clusters was also clear but slightly different than for the neritic region (Figs 7–9). The lowest amphipod abundance occurred in cluster A1, characteristic of 2003, with dominance by *V*. *armata*, *Platyscelus serratulus*, *L*. *bengalensis*, and *E*. *minuta* (GM of 12, 3, 2, and 2 ind/1000 m^3^ respectively). The main contribution to similarity was from *V*. *armata* (45.6%). *P*. *brevidens*, important in other clusters, was absent in cluster A1, and *L*. *schizogeneios* had low contribution to similarity (4.6%).

The offshore community from 2004, represented in cluster C1, was distinguished from other clusters by its high abundance of *A*. *blossevillei* (GM = 17 ind/1000 m^3^), which together with *L*. *schizogeneios*, *E*. *minuta*, and *V*. *armata* contributed 38.8% to similarity (Fig 9). Although diversity was high in cluster C1, cluster C2 had a higher number of species (44 in C1 versus 68 in C2), although this is also due to a higher number of stations aggregated in C2. The large cluster C2 may be considered characteristic of the oceanic region by the inclusion of several years (2002, 2005, 2006, and 2008; Figs 7 and 8). The most representative species were *V*. *armata*, *L*. *schizogeneios*, *E*. *minuta*, and *P*. *brevidens* (accounting for 48.8% of similarity). As can be seen, cluster C2 was relatively similar to C1, with the main disparities being a higher abundance of *V*. *armata* (54 versus 13 ind/1000 m^3^) and *P*. *brevidens* (22 versus 7 ind/1000 m^3^), and lower abundance of *A*. *blossevillei* (3 versus 17 ind/1000 m^3^).

**Fig 10:**
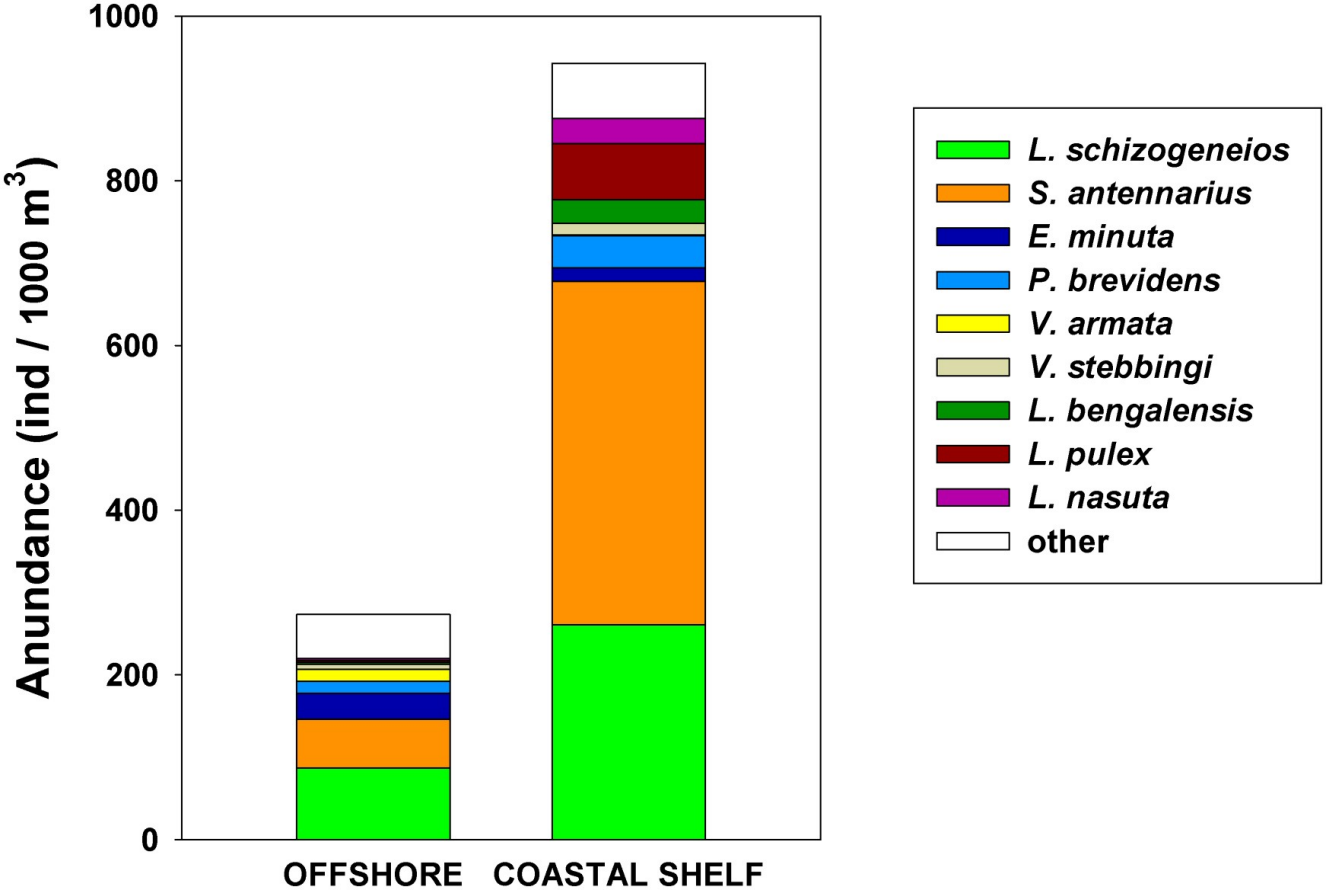
Species composition in September 2007. Stacked geometric means of the main species in the coastal shelf and offshore region.

In contrast, during September 2007 certain species not observed or scarce in the summer cruises analyzed above (e.g., *Lycaea pulex*, *L*. *nasuta Lestrigonus bengalensis*, *V*. *stebbingi*) were abundant in neritic waters (Fig 10). Dominant species such as *S*. *antennarius*, and *L*. *schizogeneios* also reached high abundances (GM 416 and 261 ind/1000 m^3^ respectively). In the offshore region, *S*. *antennarius* and *L*. *schizogenios* were also dominant but an order of magnitude lower (GM of 59 and 87 ind/1000 m^3^ respectively).

### Correlation of hyperiids and gelatinous plankton

**Fig 11:**
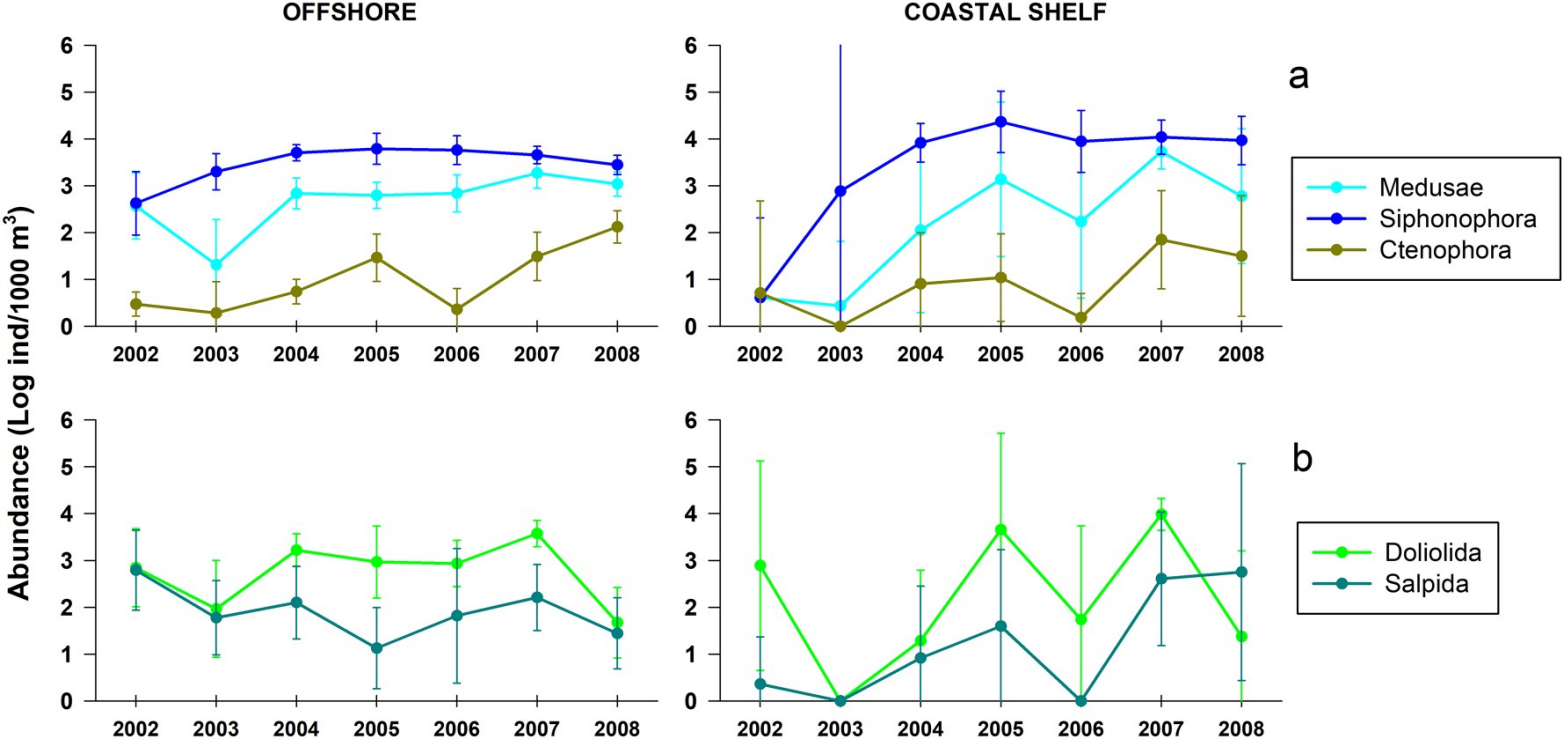
Gelatinous zooplankton in the offshore region and coastal shelf. Mean (± 95% confidence interval) of cnidarian and ctenophores (a), and tunicates (b).

Interannual changes in gelatinous organism abundances paralleled changes in hyperiid populations (Table 3). The low hyperiid abundances observed during 2003 corresponded with low abundances of medusae, ctenophores, and doliolids in the offshore region. Abundances of medusae and doliolids were significantly different in the coastal shelf than offshore (Fig 11). Differences in abundances of siphonophores and salps are also significant at p < 0.05. Low siphonophore abundance occurred in 2002 but remained at consistent levels from 2003–2008 in both regions (Fig 11a). In the coastal shelf, siphonophore abundance changed by several orders of magnitude between 2002 (GM: 3 ind/1000 m^3^) and all other years in the time-period (GMs: 765–23,051 ind/1000 m^3^). In the offshore region, differences were moderate, with 421 ind/1000 m^3^ in 2002 compared to 1,992–6,130 ind/1000 m^3^ in other years.

**Table 3:** Interannual comparisons of gelatinous groups. Abundance of gelatinous groups compared with the Kruskal-Wallis test.

Taxa	H	p	Multiple comparisons
**Offshore region** (N = 70)			
Medusae	22.8	**<0.001**	2003 < other years
Siphnophora	28.5	**<0.001**	2002 < (2004,2005,2006, 2007, 2008)
Ctenophora	38.8	**<0.001**	(2002, 2003,2006) < (2005,2007,2008) 2004 < 2008
Doliolida	28.6	**<0.001**	2008 < (2004,2005,2007) 2003 < 2007
Salpida	12.4	0.053	
All gelatinous organisms	16.1	0.013	
**Coastal shelf** (N = 38)			
Medusae	19.3	**0.004**	(2002,2003) < (2005, 2007)
Siphnophora	15.9	0.014	
Ctenophora	11.8	0.068	
Doliolida	19.6	**0.003**	2003 < (2005,2007)
Salpida	15.7	0.015	
All gelatinous organisms	12.6	0.050	

Taxa with significant results are highlighted (α < 0.01). Specific years with significant differences are indicated, which resulted from Tukey multiple comparisons.

Other gelatinous groups showed large interannual and regional variability. Medusae had particularly high abundance in the GU in 2007. Lowest abundance in both regions occurred in 2003 (GM of 20 and 2 ind/1000 m^3^ at offshore and GU, respectively; Fig 11a, Table 3). In the offshore region, ctenophores were the least abundant gelatinous group throughout the study period but increased in 2005, 2007, and 2008 compared to 2002, 2003 and 2006 (Table 4). No significant differences were observed for ctenophores in the GU.

**Table 4:** Correlation between hyperiids and potential gelatinous hosts. Spearman correlation between abundances of hyperiid amphipod species and gelatinous zooplankton groups.

Hyperiid Species	Medusae	Siphonoph.	Ctenophora	Doliolida	Salpida
*Eupronoe maculata*	0.228	-0.103	0.133	0.143	**0.405**
*Eupronoe minuta*	0.174	-0.023	**0.389**	0.225	0.193
*Hyperioides longipes*	0.217	0.216	0.084	**0.398**	0.096
*Hyperoche medusarum*	**0.455**	0.175	**0.471**	0.173	0.184
*Laxohyperia vespuliformis*	**0.540**	**0.345**	**0.442**	**0.371**	**0.400**
*Lestrigonus schizogeneios*	**0.445**	0.126	**0.406**	**0.437**	0.309
*Lycaea nasuta*	**0.352**	0.200	0.220	**0.320**	0.246
*Lycaea pulex*	**0.344**	0.126	0.159	0.264	**0.342**
*Lycaea serrata*	0.230	-0.010	0.134	0.121	**0.366**
*Oxycephalus clausi*	**0.409**	0.268	**0.376**	**0.341**	0.310
*Platyscelus ovoides*	**0.313**	0.011	0.114	**0.360**	**0.355**
*Platyscelus serratulus*	0.245	0.125	0.288	0.172	**0.322**
*Primno brevidens*	**0.383**	0.285	**0.457**	0.179	0.019
*Scina tullbergi*	0.296	0.120	**0.507**	0.075	0.006
*Simorhynchotus antennarius*	**0.455**	0.209	**0.430**	0.285	**0.355**
*Tryphana malmi*	0.305	0.170	**0.357**	0.112	0.226
*Vibilia stebbingi*	**0.479**	0.222	0.305	**0.480**	0.292

Significant correlations are highlighted (α < 0.001). Species without significant results are not shown.

Tunicates presented significant interannual differences in the GU, but tendencies were different for doliolids and salps (Fig 11b, Table 3). Doliolids were absent in 2003, and their abundance in 2002 (GM = 772 ind/1000 m^3^) was significantly lower than in 2005 and 2007 (4,528 and 9,610 ind/1000 m^3^, respectively). Salps showed higher abundances in 2007–2008 (GM of 404–562 ind/1000 m^3^) but significant differences with the Tukey test were limited to 2006 and 2008 (p = 0.036). In the offshore region, doliolids displayed lower variability compared to the strong fluctuations in their abundance in the GU. Patterns of salp abundance showed variation between regions, with high abundance during 2002–2004 in the offshore region but low abundance in the GU. In contrast, salps were more abundant in the GU during 2007–2008.

Correlation analysis between gelatinous groups and the 50 most frequent hyperiid species returned significant for 17 species (p < 0.001, Table 4; 24% with dominant, 35% common, and 41% sparse species). Notably, *L*. *vespuliformis* presented significant correlations with all five gelatinous groups. Four other species (*L*. *schizogeneios*, *O*. *clausi*, *Platyscelus ovoides*, and *S*. *antennarius*) correlated positively with three gelatinous groups, but the remaining species only correlated with one or two gelatinous groups (Table 4).

The highest number of significant correlations (p<0.001) was with medusae and ctenophores (56% of the total). These included 4 species correlated with medusae (*V*. *stebbingi*, *L*. *nasuta*, *L*. *pulex*, and *P*. *ovoides*), 3 species with ctenophores (*S*. *tullbergi*, *E*. *minuta*, and *T*. *malmi*), and 7 species correlated with both medusae and ctenophores (*Hyperoche medusarum*, *L*. *vespuliformis*, *L*. *schizogeneios*, *P*. *brevidens*, *O*. *clausi*, and *S*. *antennarius*). There was no apparent taxonomic preference for one of the two groups, and all superfamilies had one or more species correlated with both medusae and ctenophores (Table 4).

Tunicates comprised 41% of significant correlations (p<0.001), with 5 species correlated with doliolids (*V*. *stebbingi*, *H*. *longipes*, *L*. *schizogeneios*, *L*. *nasuta*, and *O*. *clausi*), 5 correlated with salps (*Eupronoe maculata*, *L*. *pulex*, *L*. *serrata*, *P*. *serratulus*, and *S*. *antennarius*), and 2 species with both tunicate groups (*L*. *vespuliformis* and *P*. *ovoides*). All superfamilies excepting Scinoidea had some correlation with tunicates (Table 4). The siphonophores only presented one significant correlation, with *Laxohyperia vespuliformis*.

## Discussion

### Interannual variability and the weak El Niño events

The region located south of Punta Eugenia is the CCS region most strongly influenced by tropical biota. Some tropical species are resident, and some appear in the region during El Niño events [1, 52]. Despite high diversity of hyperiid amphipods found in both the Gulf of Ulloa and the offshore region, their abundances were low in the present study compared to other latitudes. Total hyperiid abundance showed summer GM of 32–273 ind/1000 m^3^ during 2002–2008. In contrast, a GM of 212–867 ind/1000 m^3^ was estimated for the same period off northern Baja California (30–32°N), [3]. The low amphipod abundance found in the GU could be related to El Niño events, which appeared to have a higher impact south of Punta Eugenia, based on changes in euphausiid populations [52]. Comparing the interannual variability observed off north Baja California [3], changes at the species level were out of phase between the northern and southern Baja California regions. For example, the most abundant species, *L*. *schizogeneios*, had a GM of <1 ind/1000 m^3^ in the present study for the oceanic region during summer 2003, while off northern Baja California its GM was 75 ind/1000 m^3^ [3]. The same situation occurred for *P*. *gracilis*, with high abundance (GM = 77 ind/1000 m^3^) in the northern region during 2003 [3] but low abundances off the GU (0.6 ind/1000 m^3^). The abundance of *P*. *gracilis* is in general low off the GU compared to northern Baja California (<3 ind/1000 m^3^ across the period 2002–2008). The low abundance during 2003 off the GU was also observed in eleven other species (Table 2), suggesting a more severe effect of El Niño 2002–2003 in the southernmost region of the CCS [50, 51].

It is interesting to note that the low hyperiid abundance recorded in 2003 south of Punta Eugenia was similar to the decrease that occurred during 2005 off northern Baja California [3]. In both cases, decreases were due largely to decreased abundance of *L*. *schizogeneios* and to a lesser extent *P*. *brevidens*. The question of why these decreases occurred in different years from region to region may be answered by the occurrence of two short El Niño events, one in Jun 2002–Feb 2003 [53] and other in Jul 2004–Apr 2005 [54]. Both El Niño events were weak, but they evolved differently. The development of El Niño 2002–2003 was combined with a subarctic water intrusion coming from the north, which cooled the northern region off Baja California but not the southern region. The presence of a cyclonic eddy in the latitude of 27–29°N [50, 55] retained the cool subarctic water, helping to maintain population densities of typical California Current species north of Punta Eugenia [51].

In contrast, El Niño 2004–2005 had no simultaneous subarctic water intrusion. El Niño 2004–2005 appears to have affected all regions of the CCS mainly in autumn and winter [54, 56], with different regions experiencing these impacts at different times within those seasons. El Niño 2004–2005 first affected the GU during summer 2004. Species such as *A*. *blossevillei* and *L*. *vespuliformis*, with modest abundances in northern Baja California [3], have relatively more importance in the hyperiid community off the GU. *A*. *blossevillei* was particularly abundant in July 2004, comprising 11% of total hyperiids in that year. This species was also found in high abundance in the northern Baja California region but in January 2005 [1], associated with oligotrophic oceanic waters. Because the warm anomalies associated with El Niño spread from south to north, there was more evidence of this event during July 2004 in the oceanic region off the GU (present study) and in January 2005 off northern Baja [1]. El Niño 2004–2005 was not a predicted event [54] and is still under discussion for its occurrence and magnitude of surface water advection [57], but the hyperiid findings from the present and previous studies [1, 3] suggest a south-to-north advection of *A*. *blossevillei*.

There are no studies documenting the effects of El Niño on hyperiids or other zooplankton during 2004–2005. However, off Oregon, high proportion of warm–water neritic copepods were noted in the summer 2004, attributed to northward advection of warm water [58]. El Niño effects on higher trophic levels in the eastern Pacific are better studied and appear to relate to availability of food. For example, California sea lions (*Zalopus californicus*) performed longer foraging trips into oceanic waters during summer 2004 [59]; the Galapagos fur seal (*Arctocephalus galapagoensis*) migrated to the coast of Ecuador in September 2004 [60]; and the intertidal black sea urchin (*Tetrapygus niger*) recorded dietary changes between August 2004 and January 2005 [61].

### Cross-shelf differences in species assemblages and La Niña

Hyperiid amphipods present clear cross-shelf differences, as was established first for the California Current off Oregon [18], and further confirmed off Baja California [1]. In the present study, the influence of coastal upwelling off Punta Abreojos was consistently observed during all summers between 2002–2008 (Figs 2 and 7). Upwelling plumes spread to the south in the oceanic region and, due to the topography of the area, produce an inshore-offshore split, isolating the GU, which maintains warmer temperature. Summer enhancement of the poleward coastal current also increases temperatures inside the GU [26]. The upwelling front appears to be an effective barrier against the entry of amphipods from the open ocean to the coastal shelf.

The low frequency and abundance of hyperiids in the GU was almost constant across all summers in the period 2002–2008, as well as in other seasons during 2005 [1]. A clear exception to this pattern was the summer of 2007, when high abundances of *S*. *antennarius*, *L*. *schizogeneios*, and *L*. *pulex* were observed. This could be attributed to the influence of La Niña 2007–2008 [62], promoting enhanced biological productivity. High values of primary productiviton were recorded from January to late spring 2007 in the GU [23].

However, the increase of amphipods in the GU and the offshore region during summer 2007 could also result from a seasonal effect due to sampling in later summer (one month later than the rest of summers analyzed in the present study). Samples from 2007 likely show processes more typical of autumn or late summer, when there is a seasonal increase in hyperiid abundance and diversity off Baja California [1, 2]. The trend toward increasing abundance during autumn was first observed in inshore waters off Oregon for some species (*Hyperoche medusarum*, *Themisto pacifica*, and *Hyperia medusarum*) in 1963, and for *P*. *gracilis* in 1965 and 1966 [18]. However, *Hyperia medusarum* increased during winter 1967 [18]. In Sagami Bay, Japan, maximum abundance during the year occurred in September for 15 of 25 species, while six species increased in November [39]. Alternatively, both the seasonal and interannual influences could be the cause of increased hyperiid abundance during 2007 in the GU.

### Presence of gelatinous organisms enhances hyperiid abundance

The combination of seasonal and interannual effects, as well as the availability of abundant gelatinous organisms in 2007, could explain the high abundance of hyperiids in the coastal shelf of Baja California. Many amphipod species were significantly correlated with medusae and ctenophores (Table 4), which is consistent with symbiotic or parasitoid records in the literature [42, 63, 64]. For example, *Hyperoche medusarum* has been reported in symbiotic association with the hydromedusae *Tima Formosa* [65], *Chromatonema erythrogonon* [66], *Mitrocoma cellularia* [43], and with the ctenophores *Pleurobrachia bachei* [67], *Beroe ovata*, and *Mnemipsis leidyi* [68]. Another example is *L*. *schizogeneios*, reported as symbiont of hydromedusae *Clytia hemisphaerica*, *Liriope tetraphylla* [69], *Aequorea* sp. [63], *Leuckartiara zacae* [43], and the ctenophore *Lampea pancerina* [70].

*Hyperoche medusarum* and *L*. *schizogeneios*, as well as other species of the families Hyperiidae and Lestrigonidae, have many records of hyperiid-host associations, mainly involving medusae and ctenophores [63]. This is consistent with the positive correlations of *H*. *medusarum*, *L*. *vespuliformis*, and *L*. *schizogeneios* with medusae and ctenophores in the present study. *L*. *vespuliformis* is the only one of these three species without previous records of symbiotic associations, because it is a new species recently described [16] from the northeast Pacific near the study region. *L*. *vespuliformis* has been found in a few other locations, all in tropical-subtropical latitudes [1, 3, 17, 41]. This species was considered rare [41], but in the present study it occurred frequently and showed correlations with all five gelatinous organisms. Therefore, it is highly probable that *L*. *vespuliformis*, which is morphologically like *H*. *medusarum*, may have a symbiotic association with one or more gelatinous species, as suggested by the results of the current study.

Other abundant species in the GU during 2007 were *L*. *pulex* and *S*. *antennarius*, which both showed significant correlation with medusae and salps; *S*. *antennarius* also showed correlation with ctenophores. *L*. *pulex* has been reported in association with diverse salp species [71]. *S*. *antennarius* has only two symbiotic records with hydromedusae, one with *Geryonia proboscidalis* in the Mediterranean [42], and the other with *Liriope tetraphylla* in Monterey Bay, California [64]. Both medusae species were present in the GU during the summer of 2007 (S2 Table).

## Conclusions

In conclusion, the study region is less populated with hyperiid amphipods compared to northern Baja California. The transition zone species present in northern Baja California and other northern sectors of the CCS were also present in the GU and offshore region, but their abundances declined, particularly for *P*. *brevidens* and *P*. *gracilis*, while *S*. *antenarius* increased. During summer, contrasting cross-shelf differences in species assemblages were observed with high interannual variability. Active upwelling off Punta Abreojos forms a plume separating the inshore and offshore regions, preventing entry of amphipods to the coastal shelf. Changes in temperature and the proliferation of gelatinous organisms during La Niña 2007–2008 promoted the occupancy of the GU by hyperiids.

## Supporting information

S1 Table Oceanographic stations. Location and sampling date of oceanographic stations during the IMECOCAL cruises at the Gulf of Ulloa and offshore region.
(XLSX)

S2 Table Hyperiid species list. Hyperiid species separated by frequency of occurrence (FO) in dominant (>55%), common (31–55%), sparse (10–30%), and rare (<10%) in 108 samples analyzed. The Geometric Mean (GM) and the mean are also shown, combining data of samples collected during nighttime (N = 88). (*) indicate species found in daytime samples only.
(XLSX)

S3 Table Medusae abundance. Abundance of two species of limnomedusae during the summer of 2007 (IMECOCAL cruise 0708).
(XLSX)

S1 Fig Summertime abundance of selected species from the Gulf of Ulloa, Baja California.—Part 1. Mean (± 95% confidence interval) in the offshore and onshore regions for species in the infraorders Physososomata (a) and Physocephalata (b–f): families Scinidae (a), Paraphronimidae (b), Vibilidae (c), and Phrosinidae (d–f).
(JPG)

S2 Fig Summertime abundance of selected species from the Gulf of Ulloa, Baja California.—Part 2. Mean (± 95% confidence interval) in the offshore and onshore regions for species in the infraorder Physocephalata: families Hyperiidae (a–b), Lestrigonidae (c–d), and Phronimidae (e–f).
(JPG)

S3 Fig Summertime abundance of selected species from the Gulf of Ulloa, Baja California.—Part 3. Mean (± 95% confidence interval) in the offshore and onshore regions for species in the infraorder Physocephalata: families Eupronoidae (a–b), Platyscelidae (c–d), and Lycaeidae (e).
(JPG)
