# Supplementary figures and images for: Hyperiid amphipods from the Gulf of Ulloa and offshore region, Baja California: The possible role of the gelatinous zooplankton as a transport vector into the coastal shelf waters
Source: PLoS One. 2020 Nov 5;15(11):e0233071. doi: 10.1371/journal.pone.0233071 (PMC7643982; doi:10.1371/journal.pone.0233071)

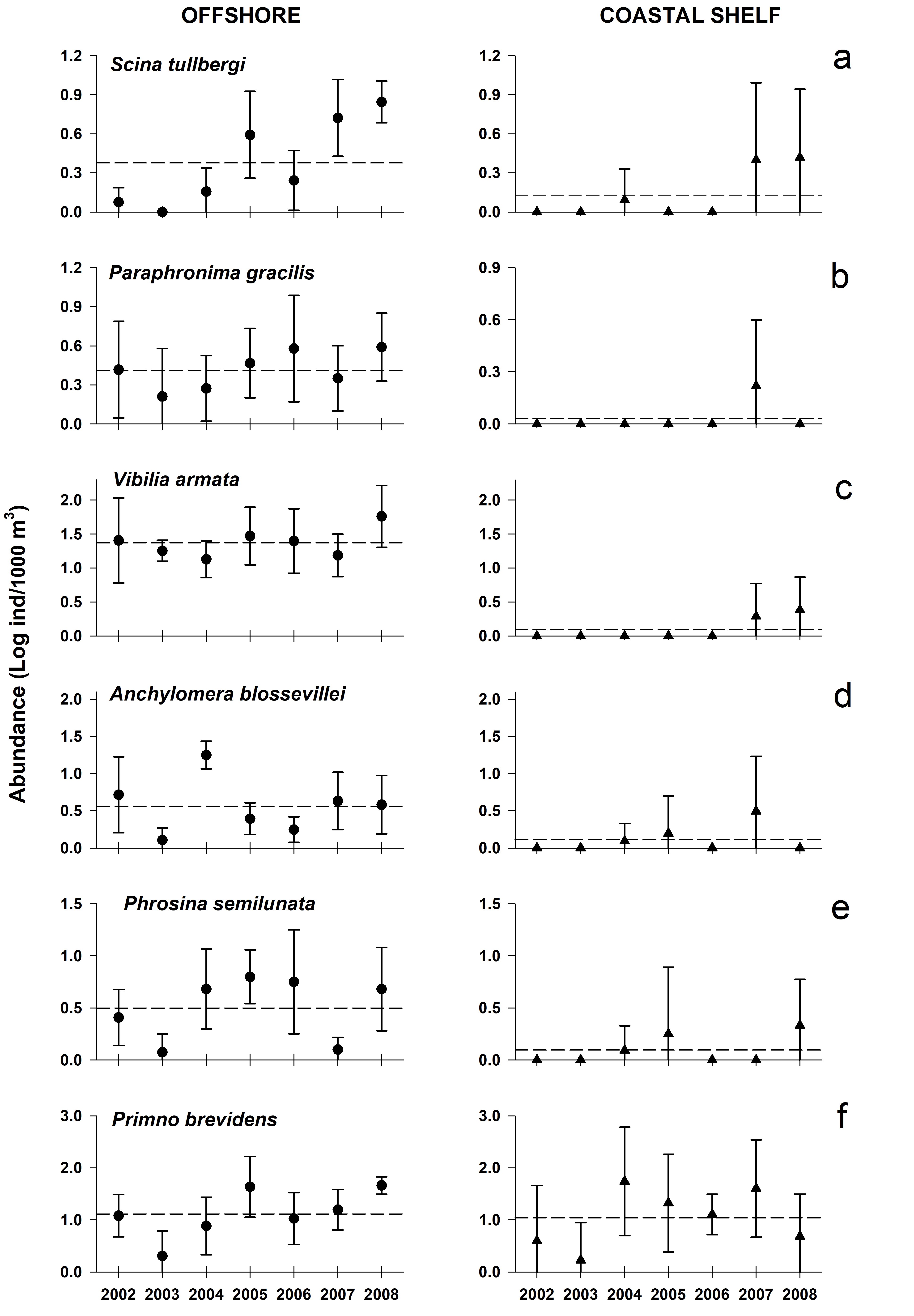

Supplement: S1 Fig — Mean (± 95% confidence interval) in the offshore and onshore regions for species in the infraorders Physososomata (a) and Physocephalata (b–f): families Scinidae (a), Paraphronimidae (b), Vibilidae (c), and Phrosinidae (d–f). (JPG) [file pone.0233071.s004.jpg]

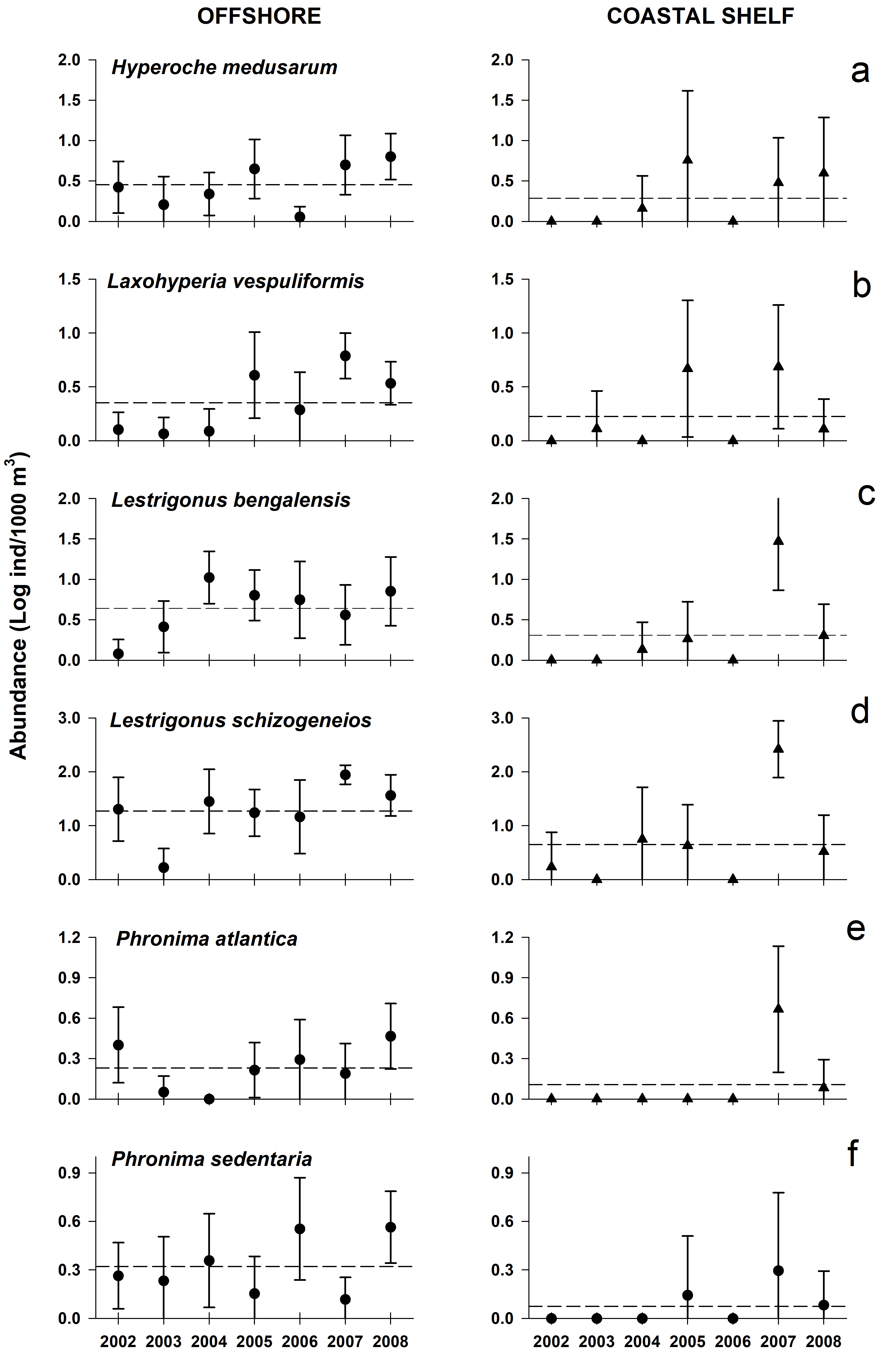

Supplement: S2 Fig — Mean (± 95% confidence interval) in the offshore and onshore regions for species in the infraorder Physocephalata: families Hyperiidae (a–b), Lestrigonidae (c–d), and Phronimidae (e–f). (JPG) [file pone.0233071.s005.JPG]

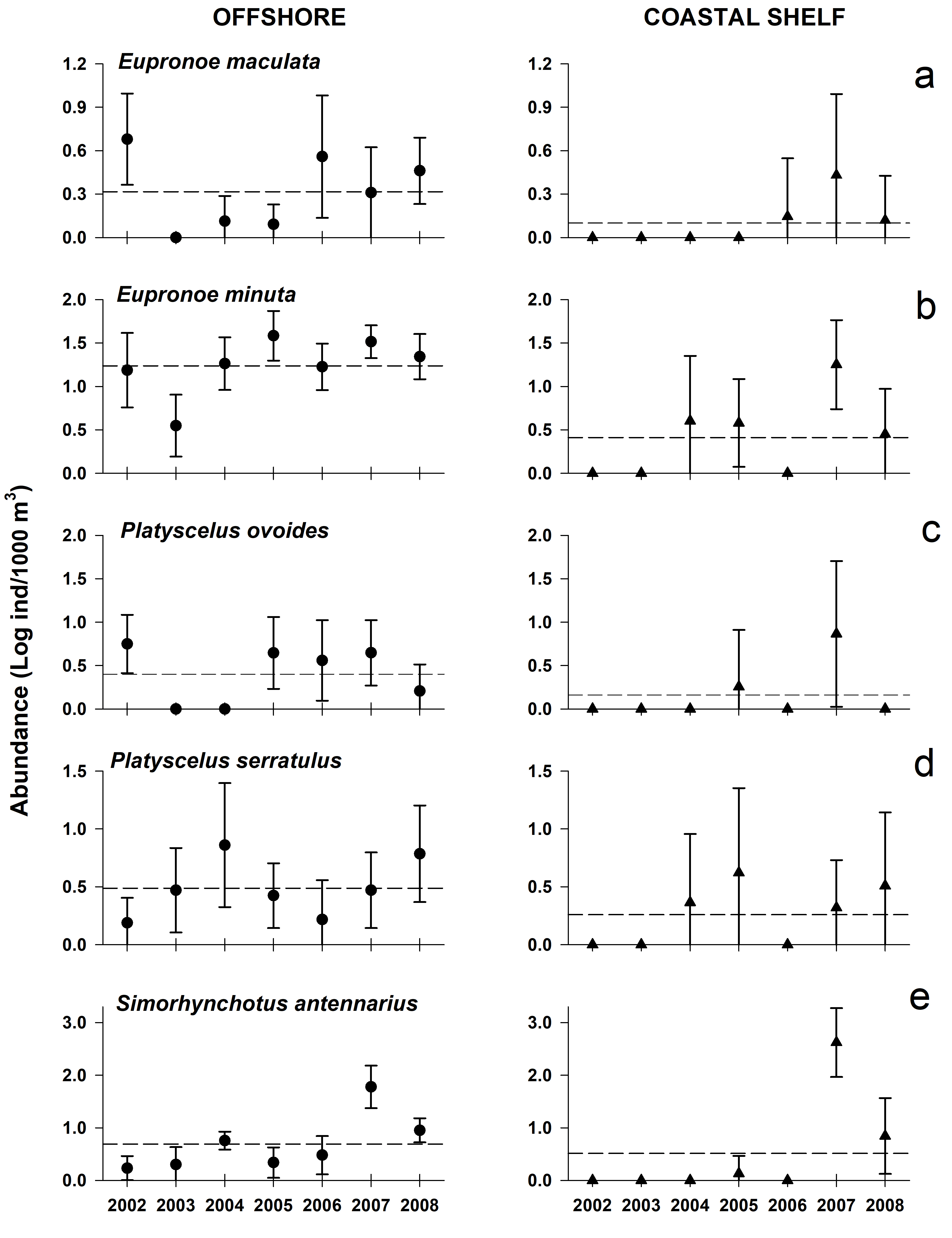

Supplement: S3 Fig — Mean (± 95% confidence interval) in the offshore and onshore regions for species in the infraorder Physocephalata: families Eupronoidae (a–b), Platyscelidae (c–d), and Lycaeidae (e). (JPG) [file pone.0233071.s006.JPG]
